# Neurophysiological Effects of the Anterior Cingulate Cortex on the Exacerbation of Crohn’s Disease: A Combined fMRI-MRS Study

**DOI:** 10.3389/fnins.2022.840149

**Published:** 2022-05-06

**Authors:** Ning Kong, Chen Gao, Fan Zhang, Meng Zhang, Juan Yue, Kun Lv, Qi Zhang, Yihong Fan, Bin Lv, Yufeng Zang, Maosheng Xu

**Affiliations:** ^1^The First School of Clinical Medicine of Zhejiang Chinese Medical University, Hangzhou, China; ^2^Department of Radiology, The First Affiliated Hospital of Zhejiang Chinese Medical University (Zhejiang Provincial Hospital of Traditional Chinese Medicine), Hangzhou, China; ^3^Key Laboratory of Digestive Pathophysiology of Zhejiang Province, The First Affiliated Hospital of Zhejiang Chinese Medical University (Zhejiang Provincial Hospital of Traditional Chinese Medicine), Hangzhou, China; ^4^Center for Cognition and Brain Disorders, The Affiliated Hospital of Hangzhou Normal University, Hangzhou, China; ^5^Institute of Psychological Sciences, Hangzhou Normal University, Hangzhou, China; ^6^Zhejiang Key Laboratory for Research in Assessment of Cognitive Impairments, Hangzhou, China; ^7^Department of Radiology, Huashan Hospital, Fudan University, Shanghai, China; ^8^Department of Gastroenterology, The First Affiliated Hospital of Zhejiang Chinese Medical University (Zhejiang Provincial Hospital of Traditional Chinese Medicine), Hangzhou, China

**Keywords:** Crohn’s disease, anterior cingulate cortex, brain-gut axis, functional magnetic resonance imaging, magnetic resonance spectroscopy

## Abstract

**Background:**

Crohn’s disease (CD) is characterized by repetitive phases of remission and exacerbation, the quality of life of patients with CD is strongly influenced by disease activity, as patients in the active phase experience significantly worse symptoms. To investigate the underlying mechanism of how the course of CD is exacerbated based on the bi-directionality of the brain-gut axis (BGA), we conducted a multi-modality neuroimaging study that combined resting-state functional magnetic resonance imaging (rs-fMRI) with proton magnetic resonance spectroscopy (MRS) to detect abnormalities in the anterior cingulate cortex (ACC).

**Materials and Methods:**

Clinical scales including Visual Analog Scale (VAS) and Hospital Anxiety and Depression Scale (HADS) were used to evaluate the degree of abdominal pain and mood state of participants. We made a comparison between CD patients in the active phase, the remission phase and healthy controls (HCs), not only employed the innovative wavelet-transform to analyze the amplitude of low frequency fluctuation (ALFF) but also compared the sensitivity of wavelet-transform and the traditional fast Fourier transform (FFT). Brain metabolites such as glutamate (Glu), myo-inositol (mIns) and gamma-aminobutyric acid (GABA) were also detected. Then correlation analysis was made to see whether changes in the ACC correlated with CD’s clinical symptoms.

**Results:**

CD patients in the active phase showed higher VAS scores (*p* = 0.025), the scores of anxiety and depression were also higher (all *p* < 0.05). Wavelet-transform is slightly more sensitive in the current research. Patients in the active phase exhibited higher ALFF in the left ACC and the left superior frontal gyrus, medial (SFGmed). Patients in the active phase showed increased Glu levels in the ACC than patients in the remission phase or HCs (*p* = 0.039 and 0.034 respectively) and lower levels of mIns than HCs (*p* = 0.036). There was a positive correlation between mWavelet-ALFF values of the ACC and HADS-depression scores in CD patients (*r* = 0.462, *p* = 0.006). Besides, concentrations of Glu positively correlated with mWavelet-ALFF in the ACC in all participants (*r* = 0.367, *p* = 0.006).

**Conclusion:**

Abnormal spontaneous activity and metabolic levels in the ACC were detected in CD patients in the active phase along with severer abdominal pain and worse mood state, these may contribute to the exacerbation of CD. Therefore, the ACC might be a potential neural alternative for managing the exacerbation of CD.

## Introduction

Crohn’s disease (CD), a chronic and inflammatory illness that mainly affects the gastrointestinal tract (GI), including the cardinal symptoms of abdominal pain, fatigue, and diarrhea, and its incidence is increasing globally (Ananthakrishnan et al., 2020). The course of CD is progressive but also relapsing and it varies among patients, with repetitive phases of remission and exacerbation. The unexpected active phase remains an intractable problem. Kochar et al. (2018) revealed that patients who had active CD experienced significantly greater symptoms than those in remission, by assessing eight GI domains (i.e., belly pain, constipation, diarrhea, gas, incontinence, nausea, reflux, and swallowing) using the PRO Measurement Information System (PROMIS). The quality of life (QoL) of CD patients was affected by disease activity strongly, the QoL of patients in the active phase was lower when compared with patients in the remission phase (Cioffi et al., 2020). The etiology of CD is multifactorial, including genetic, environmental, and immune factors, but the factors that influence the occurrence of exacerbation remains unclear. Current treatments of CD take effect by reducing the hyperactivity of the immune system, involving two phases named induction and maintenance to achieve remission (Cushing and Higgins, 2021). However, a radical cure for CD has not been found yet. Hence, determining how the remission phase changes into the active phase, and the underlying factors that exacerbate the clinical course of CD is critical and has great clinical significance.

Recent findings have highlighted the activity of the brain-gut axis (BGA) in the pathogenesis of CD. The BGA is an invisible, connection between the gut and the brain that is bi-directional, and consists of the central nervous system, the enteric nervous system, the hypothalamic–pituitary–adrenal (HPA) axis as well as the autonomic nervous system, which has been demonstrated to contribute to CD’s psychiatric comorbidities, including depression and anxiety, and the development of abdominal pain syndromes (Mayer and Tillisch, 2011; Gracie et al., 2018). Brain activity and the course of CD are intimately related. In CD, from a gut to brain perspective, higher levels of circulating cytokines could cross the blood-brain barrier, inducing oligodendrocyte, astrocyte apoptosis and neuroinflammation, which results in multiple kinds of abnormalities in the brain (Miller et al., 2009; Abautret-Daly et al., 2018). To examine the CD-induced changes in brain regions and determine their microstructure and interactions, neuroimaging is a valuable technique that is non-invasive and is able to have real-time detections, such as resting-state functional magnetic resonance imaging (rs-fMRI) as well as proton magnetic resonance spectroscopy (MRS). The latter is able to numerically quantify metabolites and neurotransmitters, including creatine (Cr), glutamate (Glu), myo-inositol (mIns), gamma-aminobutyric acid (GABA), and *N*-acetylaspartate (NAA). The findings are commonly reported as ratios in relation to Cr (Squarcina et al., 2017). The basic premise of rs-fMRI is to use blood-oxygen-level-dependent (BOLD) imaging that based on detection of hemodynamic responses to changes of spontaneous fluctuations or neural metabolism when the subject is not engaged in an active task. Numerous neuroimaging studies of patients with CD have demonstrated changes in the brain, mostly by comparing patients in the remission phase with normal controls (Bao et al., 2018; Nair et al., 2019). Moreover, previous studies on the changing phases of CD have mostly focused on quantitative assessments of the GI tract or the gut microbiome, other than potential brain abnormalities; most of the researches were conducted based on mono-modality imaging that only covers sMRI, fMRI or MRS, a multi-modality research is infrequent though it may better reflects the underlying neurophysiological effects of specific brain areas (Becker et al., 2021; Cococcioni et al., 2021). Abulseoud et al. (2020) combined the use of rs-fMRI with MRS to investigate functional activity in the human brain to investigate the effects of short-term nicotine deprivation, considering that compared with using task-induced BOLD signal changes, the time needed for resting-state detections of BOLD signals is more similar to that needed for acquiring the relatively static Glu in MRS (Abulseoud et al., 2020). Zhang et al. (2016) found the altered amplitude of low frequency fluctuation (ALFF) values in medial prefrontal cortex (mPFC) correlated positively with regional levels of glutamate in female patients with depression. Delli Pizzi et al. (2017) found that the GABA+ levels within the mPFC and the functional connectivity (FC) between amygdala and ventromedial PFC correlates negatively. Therefore, these studies showed that a combined fMRI-MRS study can provide more pathological information within the region of interest.

The anterior cingulate cortex (ACC), which has been proved to be a component of the limbic system, functions as a relay hub and transmits input signals after evaluating the requirements from other regions to guide adaptive behaviors, also plays an important component in not only the salience network but also the default mode network (Tsai et al., 2020; Tu et al., 2021). Matisz and Gruber (2022) regarded ACC as a primary target in most gastrointestinal diseases and disorders (GIDD) as this region is more sensitive to metabolic stress than others, and the neuroimmune processes around are more impactable, besides, it relates to the unique activity-dependent learning mechanisms. It is common knowledge that patients in the active phase of CD have higher levels of inflammatory cytokines than patients in the remission phase, and it’s noteworthy that the ACC is sensitive to cytokines particularly (Miller et al., 2013), which suggests that the ACC may be more vulnerable to inflammatory attacks. In a previous review we published, we summarized changes of the ACC in CD patients and found that the changes had covered multiple facets, including function, structure, and metabolism (Kong et al., 2021), according with Matisz’s idea. In addition to CD, ulcerative colitis (a form of inflammatory bowel disease) and other BGA-related diseases (e.g., irritable bowel syndrome and functional constipation) have also been found to associate with ACC anomalies (Wang et al., 2017; Fan et al., 2019; Liu et al., 2021). Hence, we saw the ACC as a promising candidate for our research.

Our study is to focus on the effects of brain abnormalities on the CD’s alternating phases. We hypothesized that the changing phases of CD (the active phase and the remission phase) would be associated with neurochemical and functional interactions within the ACC.

## Materials and Methods

### Subjects

The prospective study protocol was approved by the Ethics Committee of the First Affiliated Hospital of Zhejiang Chinese Medical University. Initially 63 participants including 42 patients and 21 HCs were recruited from the Department of Gastroenterology of the First Affiliated Hospital of Zhejiang Chinese Medical University and through local advertising, all the participants signed written informed consent forms. The inclusion criteria were: (1) having a diagnosis of CD confirmed prior to the study by an endoscopic and/or histological examination; (2) being right-handed; (3) being 18 to 60 years old; and (4) having more than 9 years of education. The exclusion criteria were: (1) patients who had a previous history of, or currently had a neurological disease, a psychiatric disease, head trauma, or with consciousness loss; (2) patients using opioids, corticosteroids or psychotropic drugs during the past 3 months; (3) patients with claustrophobia; (4) patients with metal implants; or (5) women who were currently menstruating, pregnant, or lactating.

**FIGURE 1:**
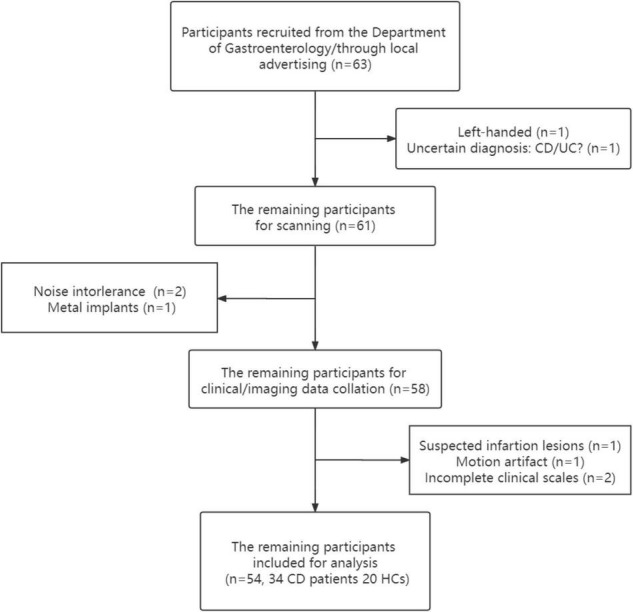
Flow chart of recruitment.

The Harvey-Bradshaw Index (HBI), which was used to evaluate disease activity, was scored by a gastroenterological expert. The HBI is a simpler substitute for the Crohn’s Disease Activity Index (CDAI), and it is less cumbersome than the CDAI in clinical practice; a score ≤ 4 corresponds to a CDAI score ≤ 150 (clinical remission) (Vermeire et al., 2010). The CD patients were assigned to remission group (RG) and active group (AG) based on their HBI scores. The abdominal pain of patients with CD was measured using a Visual Analog Scale (VAS), and anxiety and depression state among all the subjects were measured using the Hospital Anxiety and Depression Scale (HADS). The study was based on a previous study which shared a part of same MRS data (Lv et al., 2018), but all the cases were re-divided in the current study. After screening and exclusion, 54 participants including 34 patients and 20 healthy controls were included in further analysis (Figure 1).

### Functional Magnetic Resonance Imaging Data Acquisition and Preprocessing

All the MRI, rs-fMRI and MRS data were collected using a 3 T MR scanner (Discovery 750, GE Healthcare, Milwaukee, WI, United States) in the Department of Radiology at the First Affiliated Hospital of Zhejiang Chinese Medical University. We used a custom-built head holder along with a pair of foam pads to minimize the effect of head motion. We obtained a set of high-resolution T1-weighted structural images using a 3-dimensional brain volume imaging (BRAVO) sequence: TR = 8.2 ms, TE = 3.2 ms, FA = 12 degrees, matrix = 256 × 256, slice thickness = 1.0 mm, with no gaps. The functional images were acquired employing an echo planar imaging (EPI) sequence: TR = 2,000 ms, TE = 35 ms, FOV = 256 mm × 256 mm, matrix size = 64 × 64, in-plane resolution = 3.75 mm × 3.75 mm, and slice thickness = 4 mm, FA = 90°, and slices = 30. All the subjects were told to (a) relax, (b) keep their eyes closed, (c) stay awake but don’t think about anything during the MRI examination. Preprocessing of the rs-fMRI data was performed using SPM8^1^ and RESTPlus V1.25^2^. We disposed of the first 10 volumes of data to eliminate the possible influence of the subjects adapting to the environment and the magnetic equilibrium effect. Slice timing and corrections for head motion were performed for the rest of the data. No data had to be excluded because of artifact of head motion (over 1.5 mm translation or over 1.5° rotation in any direction). Spatial normalization was conducted by using the standard EPI template. Subsequently, we used a Gaussian kernel of 6 mm full width at half-maximum (FWHM) to smooth the data spatially.

We regressed out six head motion parameters (X, Y, and Z transitions and roll, pitch, and yaw rotations), white matter signals and cerebral spinal fluid (CSF) signals to create more effective maps. ALFF will be computed across 0.01–0.08 Hz (i.e. the conventional band) to reduce the effect of very high-frequency physiological noise or low-frequency drift.

The wavelet transform, was implemented *via* RESTPlus in this study to acquire wavelet-transform ALFF. We used db2 as the mother wavelet and for standardization purposes of Wavelet-ALFF, each Wavelet-ALFF was standardized by dividing the value of each voxel’s Wavelet-ALFF by the average “global” Wavelet-ALFF to get the “mean” wavelet-ALFF (mWavelet-ALFF).

Though wavelet-transform should be more sensitive in detecting complex series theoretically, few researches had utilized this newly-developed metric in ALFF analysis as yet. To consolidate the idea that wavelet-transform can be another potent metric in localized brain activity analysis, we performed a comparison between wavelet-transform and fast Fourier transform (FFT), in terms of sensitivity. The standardized ALFF values based on FFT will be denoted as “mALFF”. The ways for acquiring mALFF values are similar to those for mWavelet-ALFF values. The comparison method refers to Luo et al. (2020): the total number of voxels that survived after multiple comparison correction will be calculated. The formula of ratio is as follows:


(1) $$r\,a\,t\,i\,o=\frac{N\,u\,m\,b\,e\,r\,W\,a\,v\,e\,l\,e\,t-A\,L\,F\,F}{N\,u\,m\,b\,e\,r\,F\,F\,T-A\,L\,F\,F}.$$

*Number* denotes the number of voxels after correction. The higher the ratio is (>1), the higher sensitivity the wavelet-transform exhibits. We would take the more sensitive one as the functional imaging marker in the subsequent analysis.

### ^1^H-MRS Data Acquisition and Processing

**FIGURE 2:**
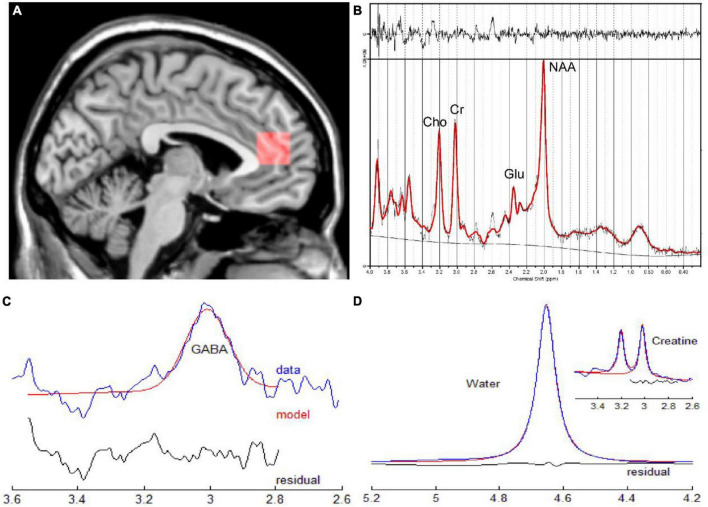
**(A)** Sagittal slice indicating the position of the ROI for single voxel spectroscopy in the ACC. **(B)** Corresponding quantitative findings of the LCModel for a male patient (33 years old) diagnosed with CD in the active phase. **(C,D)** Corresponding quantitative results of GANNET for a male patient (29 years old)diagnosed with CD in the remission phase. ACC, anterior cingulate cortex; CD, Crohn’s Disease; ROI, region of interest.

The anatomical BRAVO sequence mentioned above was used for the orientation and positioning. Point resolved spectroscopy (PRESS) was used for a single voxel spectrum. The voxel position in the bilateral ACC is shown in Figure 2A: TR = 2,000 ms, TE = 35 ms, Voxel size = 20 × 20 × 20 mm^3^, total number of scans = 64, number of excitations (NEX) = 8, water suppressed, with automatic shimmering. The pre-scan requirements were < 8 Hz in automatic shimmering at FWHM and > 95% in water suppression. In addition, the Mescher-Garwood point resolved spectroscopy (MEGA-PRESS) method was used in 5 active-CD patients, 7 remission-CD patients and 8 HCs to acquire GABA+ data: TR = 2,000 ms, TE = 68 ms, Voxel size = 20 × 20 × 20 mm^3^, total number of scans = 64, NEX = 8, water suppressed. The pre-scan requirements were identical to those used for PRESS acquisition.

The raw P files of the MRS were transferred to a workstation that was equipped with a linear combination model (LCModel) and GANNET (GABA analysis tool) for processing (Figures 2B–D). We only analyzed neurochemicals processed by LCModel with Cramer-Rao Lower Bounds < 20%. Glx refers to the sum of glutamate and glutamine. To indicate that MEGA-PRESS sequence would contain some signals from co-edited macromolecule, measurements of GABA would be reported as GABA+. The relative concentration of metabolites were assessed in this study since the reference values were tCr values.

### Statistical Analysis

Ages and clinical scores including the HADS-A and HADS-D scores among three groups were compared by using one-way analysis of variance (ANOVA), and the results are presented as mean ± standard deviation. Wilcoxon rank-sum test was conducted to compare the VAS scores and the duration between the patient groups, results are described as median (interquartile range). Gender differences were compared using chi-squared test. Group differences were considered to be statistically significant at *p* < 0.05.

ANOVA tests with Bonferroni-corrected *post hoc* analysis were conducted on the LCModel quantification data and the GABA+ data to test the hypothesis that the metabolites among the three groups differ, *p* < 0.05 was set as the level of statistical significance. SPSS software (version 25.0) was used. ANOVA was used to analyze whole brain inter-group differences of mWavelet-ALFF/mALFF values *via* DPABI (edition 4.3), with least significant difference (LSD) selected for *post hoc* tests. Then the resultant maps were corrected by using Gaussian random field (GRF) correction (voxel threshold of *P* < 0.005, cluster threshold of *P* < 0.05). We conducted Spearman correlations on metabolite concentrations/the functional imaging marker and clinical scores to determine the association between brain abnormalities and CD symptoms. In addition, Pearson correlations were used to determine the association between metabolite concentrations and the imaging marker’s values. To ensure that we would only analyze the ALFF values that were from the voxels whose positions overlap with those in MRS orientation, we created a 20 × 20 × 20 mm^3^ mask that covered the same ACC part in the MRS data acquisition procedure, put it in the resultant maps and only extracted the values of the peak voxel inside the mask for correlation analysis. Statistical significance was set at *p* < 0.05.

## Results

### Clinical and Demographic Characteristics

The demographic and clinical variables are shown in Table 1. No significant difference was found for age, gender among the three groups. There’s no significant difference of duration between the patient groups. The HADS-A scores of the active-CD group were significantly higher than the scores of the remission-CD group and HCs (*p* = 0.009 and *p* = 0.006, respectively). Their HADS-D scores were also higher (*p* = 0.009 and *p* = 0.047, respectively), indicating worse mood state in active group patients. Moreover, the VAS scores were higher in the active-CD group than in the remission-CD group (*p* = 0.025), in accordance with the clinical manifestations of CD.

**TABLE 1:** Demographic and clinical information of the participants.

Variables	AP (*n* = 15)	RP (*n* = 19)	HCs (*n* = 20)	*p*-value	
Ages (years)^a^	30.33 ± 9.17	32.00 ± 9.84	29.35 ± 7.60	>0.05	
Gender (M/F)^b^	9/6	13/6	12/8	>0.05	
VAS^c^	3 (0, 6)	0 (0, 2)	/	*0.025	
Duration (years)^c^	2 (0.5, 10)	4 (2, 5)	/	>0.05	
HADS-A^a^	6.07 ± 3.10	3.63 ± 2.60	3.55 ± 2.09	*0.009	^#^0.006
HADS-D^a^	5.73 ± 3.92	3.05 ± 2.63	3.75 ± 1.97	*0.009	^#^0.047

*Statistically significant at the 0.05 level (two tailed). AG, active group; F, female; HADS, Hospital Anxiety and Depression Scale; HCs, Healthy controls; M, male; RG, Remission group; VAS, visual analog scale.*

*^a^One-way analysis of variance (ANOVA). Results are presented as mean ± SD.*

*^b^Chi-squared test.*

*^c^Wilcoxon rank-sum test. Results are described as median (interquartile range).*

*AG compared with RG.

*^#^AG compared with HCs.*

### Comparison of mWavelet-ALFF/mALFF Among Active Group, Remission Group and Healthy Controls

**FIGURE 3:**
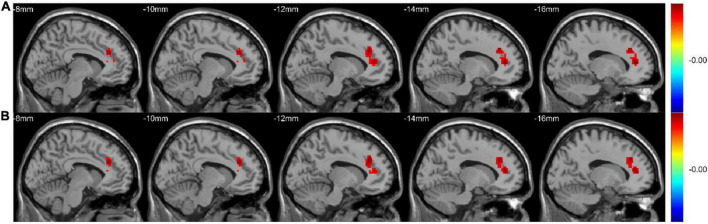
The mWavelet-ALFF/mALFF values in the left ACC and the left SFGmed were significantly higher in the active-CD group. The F map (All GRF-corrected, *p* < 0.05) of active group vs. remission group in the conventional band (0.01–0.08 Hz) by WT-ALFF **(A)** and FFT-ALFF **(B)**, respectively. ACC, anterior cingulate cortex; ALFF, amplitude of low-frequency fluctuation; FFT, fast Fourier transform; SFGmed, superior frontal gyrus, medial; WT, wavelet-transform.

The active-CD group exhibited significantly higher mWavelet-ALFF/mALFF in the left ACC and the left superior frontal gyrus, medial (SFGmed) than the remission-CD group did (GRF-corrected, *p* < 0.05; Table 2 and Figure 3). We didn’t detect other significant differences among the three groups. Based on the F map of active group vs. remission group in the conventional band, though mWavelet-ALFF and mALFF results showed similar spatial pattern, different numbers of voxels were detected (88 and 85 respectively). The ratio is about 1.04, suggesting that wavelet-transform is slightly more sensitive. We select mWavelet-ALFF as our functional imaging marker in the subsequent analysis.

**TABLE 2:** mWaveletALFF results between active and remission CD groups.

Brain region	Cluster	Peak MNI coordinate			Peak *t* value
		x	y	z	
Limbic Lobe	54				
Anterior Cingulate	48				
Frontal Lobe	34				
Medial Frontal Gyrus	25				
Cingulum_Ant_L (aal)	24	−12	42	12	3.77
Frontal_Sup_Medial_L (aal)	14				

*AAL, anatomical automatic labeling; ALFF, amplitude of low frequency fluctuation; L, left; MNI, Montreal Neurological Institute.*

### Comparison of Metabolic Levels Among Active Group, Remission Group and Healthy Controls

**FIGURE 4:**
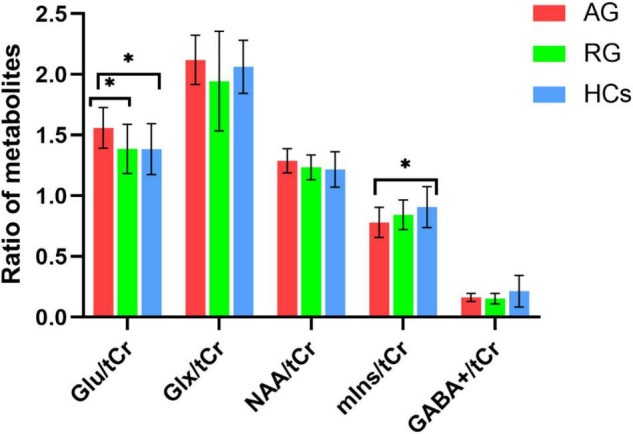
The mean ratio of brain metabolites of the three groups. CD patients in the active phase had higher Glu/tCr levels than those in remission phase (*p* = 0.039) and HCs (*p* = 0.036) in the bilateral ACC. Patients in the active phase had lower mIns/tCr than HCs (*p* = 0.036). There were no significnat differences in NAA/tCr, Glx/tCr, or GABA+ among the three groups (all *p* > 0.05). AG, active group; GABA, gamma-aminobutyric acid; Glu, glutamate; Glx, glutamate (Glu)+glutamine (Gln); HCs, healthy controls; NAA, *N*-acetyl-aspartate; mIns, myo-inositol; RG, remissive group; tCr, total creatine. “*”, statistically significant.

**FIGURE 5:**
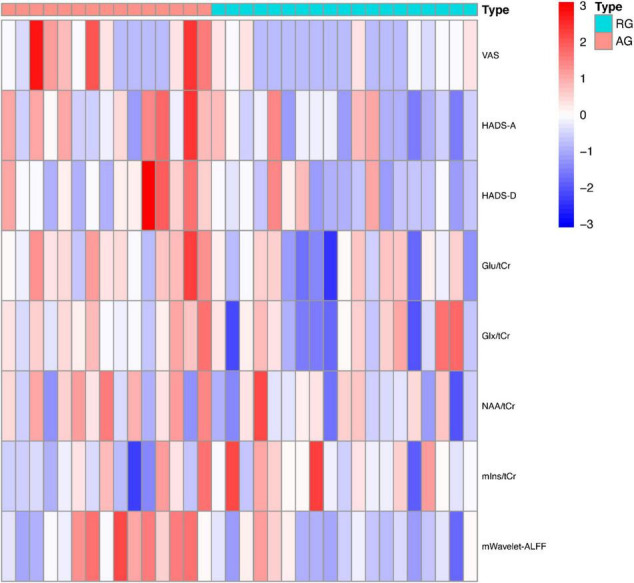
The heatmap of clinical and imaging data of CD patients. ALFF, amplitude of low frequency fluctuation; AG, active group; Glu, glutamate; Glx, glutamate (Glu)+glutamine (Gln); HADS, Hospital Anxiety Depression Scale; NAA, *N*-acetyl-aspartate; mIns, myo-inositol; RG, remissive group; tCr, total creatine; VAS, Visual Analog Scale.

ANOVA tests with Bonferroni-corrected *post hoc* analysis revealed that CD patients in the active phase had significantly higher levels of Glu/tCr than the patients in the remission phase and HCs (*p* = 0.039, *p* = 0.034) in the bilateral ACC. Patients in the active group showed lower levels of mIns/tCr than HCs (*p* = 0.036). Other differences were not statistically significant in concentrations of NAA, Glx or GABA+ (all *p* > 0.05). The results are presented in Table 3 and Figure 4. A heatmap (Figure 5) was drawn by the package “pheatmap” in R statistical software (version 4.0.5) to make a more obvious depiction of data of all the CD patients (GABA+ was not included due to small sample size).

**TABLE 3:** Mean ratio of metabolites levels in ACC among the three groups.

Ratio of Metabolites	Sample size (AG /RG/HCs)	AG	RG	HCs	*p*-value	
Glu/tCr	15/19/20	1.64 ± 0.20	1.39 ± 0.21	1.47 ± 0.26	*0.039	^#^0.034
Glx/tCr	15/19/20	2.13 ± 0.21	2.06 ± 0.48	2.14 ± 0.10	>0.05	
NAA/tCr	15/19/20	1.24 ± 0.12	1.22 ± 0.09	1.19 ± 0.14	>0.05	
mIns/tCr	15/19/20	0.76 ± 0.06	0.78 ± 0.10	0.85 ± 0.06	^#^0.036	
GABA+	5/7/8	0.16 ± 0.03	0.15 ± 0.04	0.21 ± 0.13	>0.05	

*ANOVA tests with Bonferroni-corrected post hoc analysis, statistically significant at 0.05 level (two tailed).*

*Values are presented as mean ± SD.*

*ACC, anterior cingulate cortex; AG, active group; CD, Crohn’s disease; GABA, gamma-aminobutyric acid; HCs, healthy controls; Glu, glutamate; Glx, glutamate*

*(Glu)+glutamine (Gln); NAA, N-acetyl-aspartate; mIns, myo-inositol; RG, Remission group; tCr, total creatine.*

*AG compared with RG.

*^#^AG compared with HCs.*

### Correlation of Regional Metabolic Levels/Functional Imaging Marker With Clinical Scores

**FIGURE 6:**
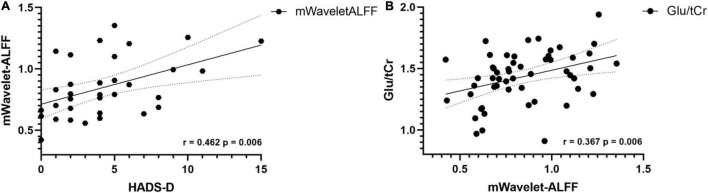
**(A)** In CD patients, the mWavelet-ALFF value in the left ACC had a positive correlation with patients’ HADS-D scores (*r* = 0.462, *p* = 0.006). **(B)** Concentrations of glutamate correlates positively with mWavelet-ALFF values in the left ACC in all participants (*r* = 0.367, *p* = 0.006). ACC, anterior cingulate cortex; ALFF, amplitude of low frequency fluctuation; Glu, glutamate; HADS, Hospital Anxiety Depression Scale; tCr, total creatine.

In terms of metabolites and clinical manifestations, our analyses found positive correlations between the VAS scores and the concentrations of Glu (*r* = 0.41, *p* = 0.016) and Glx (*r* = 0.39, *p* = 0.021) in the patient groups. However, when Bonferroni correction was employed, the correlations were no longer statistically significant. In terms of mWavelet-ALFF and clinical scores in the patient group, a Bonferroni corrected *p* value of < 0.05/3 would be considered statistically significant. A positive relation exists between mWavelet-ALFF values of the left ACC and HADS-D scores (*r* = 0.462, *p* = 0.006, Figure 6A). No other correlations of regional metabolic levels/mWavelet-ALFF with clinical scores were found to be significant.

### Correlation of Regional Metabolic Levels With the Functional Imaging Marker

The Glu concentration was found to have a positive correlation with the regional mWavelet-ALFF values of the peak voxel in the left ACC (*r* = 0.367, *p* = 0.006, Figure 6B) in all participants and the correlation survived after Bonferroni correction (*p* < 0.05/5). The correlations of the mWavelet-ALFF values with the concentrations of the other metabolites (i.e., NAA, mIns, Glx, and GABA+) were not significant.

## Discussion

Our study is the first to report ACC’s neurophysiological effects on CD’s phases, using a multimodal imaging approach that combined rs-fMRI and ^1^H MRS. We demonstrated that wavelet-transform may be slightly more sensitive than FFT in ALFF analysis. CD patients in the active phase exhibited higher spontaneous brain activity in the left ACC and the left SFGmed and also showed higher levels of Glu in the ACC. The degree of abdominal pain in the active group was also higher, and patients in the active phase showed higher scores in HADS. There was a positive relation between mWavelet-ALFF in the ACC and HADS-D scores, and the concentrations of Glu positively related with mWavelet-ALFF in the ACC.

ALFF is a classic metric that has mostly been calculated with FFT in previous studies to determine the abnormal brain activity precisely, with the energy of a time series being broken down into sets of stationary sinusoidal functions of different frequencies (Zang et al., 2007). However, since the waveforms of fMRI signals are quite complex, and they are not stationary, transient phenomena are difficult to detect using traditional FFT. Recently, Luo at al. found that the Wavelet-ALFF was generally more sensitive than those with FFT in all frequency bands, of all five mother wavelets (Luo et al., 2020). By comparison of the generated F maps, we agree with Luo’s conclusion that wavelet-based analysis is slightly more sensitive in the conventional band (i.e., 0.01–0.08 Hz) and support the idea that wavelet analysis provides a useful set of ways to analyze the characteristics of complex time series and non-stationary data, it is more effective in depicting complex waveforms that are much closer to real-life situations (Patel et al., 2014).

In the current study, patients in the active phase showed higher scores in HADS. By using the mWavelet-ALFF as the functional imaging marker, we demonstrated that when compared with patients in the remission phase, patients in active phase showed increased spontaneous brain activity in the left ACC, and the values positively correlated with HADS-D scores in all CD patients. A meta-analysis indicate that MDD patients displayed higher ALFF in ACC, Zhang et al. (2019) found there were increases in ALFF in the ACC from patients who reported having chronic lower back pain (Gong et al., 2020). Taking our results and these pain and mood-related brain changes together, they may be attributed to the role of ACC in affection regulation, visceromotor function, and emotional, somatosensory, and self-referential processing as a part of limbic system (Etkin and Schatzberg, 2011). In addition to ACC, patients in the active group also showed increased values of ALFF in the left SFGmed. As a part in frontal cortex, SFGmed is involved in emotion and cognition process. Yan et al. (2021) revealed higher regional homogeneity (ReHo) values in SFGmed in MDD patients, Zheng et al. (2020) revealed stronger FC between the left amygdala and bilateral ACC/SFGmed, along with higher levels of interleukin-6 and anxiety in patients with end stage renal disease (Bacteroides mainly). Taken together, the evidence highlights the important role SFGmed plays in mood disorders.

In the brain, Glu is the primary excitatory neurotransmitter, the excessive release of Glu is related to excitotoxicity-induced injury. In contrast, GABA is the primary inhibitory neurotransmitter. In our research, patients who are in the active phase had higher concentrations of Glu in the ACC. Higher Glu levels might indicate neuronal hyperexcitability, offering a partial explanation of higher mWavelet-ALFF/mALFF values. As a component of the “pain matrix,” ACC processes signals from visceral nociceptive afferents and exhibits long-term potentiation that affects excitatory synapses and involved in neural plasticity (Zhuo, 2020). Evidence suggests that Glu is a candidate biomarker for pain. Hansen et al. (2019) showed that patients with a high level of Glu in the ACC report more severe abdominal pain. Legarreta et al. (2021) found that in the ACC, the Glu/GABA ratio associated with anxiety and depression positively, and these mood disorders related to chronic pain may be attribute to the disturbance in the excitatory-inhibitory balance. In our previous research, we found that compared to those without abdominal pain, CD patients with abdominal pain had higher concentrations of Glu, indicating it may associate with pain severity (Lv et al., 2018). In addition to pain, altered Glu neurotransmission, along with excessive activation of inflammation, have been suggested to be important pathophysiological pathways in mood disorders. They might merge at the glial level to produce mood disorders and behavioral alterations. Interestingly, in the active group, concentrations of mIns in the ACC were found to be lower than those from HCs. Normally mIns is transiently transported into astrocytes and can be taken as a non-specific biomarker of astrocyte’s state, so we speculate decreased levels of mIns in CD patients may reflect dysfunction of glial cells (Shirayama et al., 2017).

Glu is associated with energy metabolism in the brain as an excitatory neurotransmitter. The majority of ACC neurons are glutamatergic. By conducting correlation analysis, we found the concentrations of Glu were positively related to mWavelet-ALFF values, according with previous studies. For instance, Enzi et al. (2012) demonstrated that the concentrations of Glu directly related to the level of resting-state activity in the ACC, using a combined fMRI–MRS approach with healthy subjects. Duncan et al. (2011) demonstrated in subregions of ACC, Gluconcentration correlates with interregional signal changes.

In our current research, ACC demonstrated functional along with metabolic alterations, which may be due to pain and mood state and the underlying neuroinflammation process. To improve the QoL for CD patients, though hasn’t been widely used in clinical treatment, we propose that ACC may be taken not only as a pathogenesis target, but also a therapeutic target, considering that pain, depression and anxiety are all indications for ACC treatment: Russo and Sheth (2015) suggests that deep brain stimulation of ACC can be an alternative option to treat chronic neuropathic pain Jing et al. (2020) thought ACC can be an effective region for repetitive transcranial magnetic stimulation for treatment of MDD. We hope our findings can help in providing reference for clinical prevention and treatment of CD.

Our study needs to be considered in light of its limitations and strengths. First, the study had a relatively small sample size, particularly in the GABA+ acquisition, and no significant group differences of GABA+ were found, even though theoretically, levels of GABA+ are expected to be lower as there is an excitatory-inhibitory balance in neurotransmitters. The absence of significant differences may be due to the disease itself, as it could disrupt this balance, or it may be attribute to our relatively small size of samples, so a multi-canter research that covers more samples should be conducted in the future. Second, in addition to current methods we used, structural network topology can also provide crucial information, so more multi-modality MRI analysis should be conducted in subsequent studies. Third, considering the bidirectionality of the BGA, it’s hard for our recent research to discern whether the clinical findings play as the cause or the consequence of the changes in the brain. Future studies with more clinical indicators and mediation analysis may help to define the pathway. Despite these limitations, we conducted the first study to report the exacerbation of CD from the non-invasive neuroimaging perspective, and it has helped to strengthen our understanding about the intricate brain activities underlying the alternating phases of CD.

## Conclusion

Patients with CD in the active phase had worse mood state and severer abdominal pain. The abnormal metabolite levels and spontaneous activity in the ACC correlates with CD’s clinical symptoms and may contribute to the exacerbation of CD.

## Data Availability Statement

The raw data supporting the conclusions of this article will be made available from the corresponding author upon reasonable request.

## Ethics Statement

The studies involving human participants were reviewed and approved by the Ethics Committee of the First Affiliated Hospital of Zhejiang Chinese Medical University. The patients/participants provided their written informed consent to participate in this study. Written informed consent was obtained from the individual(s) for the publication of any potentially identifiable images or data included in this article.

## Author Contributions

NK and CG: data curation, investigation, methodology, formal analysis, writing—original draft, and writing—review and editing. FZ: methodology, software, validation, and writing—review and editing. MZ and JY: methodology, software, validation, and visualization. KL: data curation, methodology, and software. QZ: methodology and formal analysis. YF: conceptualization, data curation, investigation, and supervision. BL: funding acquisition, resources, and supervision. YZ: methodology, resources, and supervision. MX: conceptualization, funding acquisition, investigation, methodology, project administration, supervision, resources, and writing—review and editing. All authors contributed to the article and approved the submitted version.

## Conflict of Interest

The authors declare that the research was conducted in the absence of any commercial or financial relationships that could be construed as a potential conflict of interest.

## Publisher’s Note

All claims expressed in this article are solely those of the authors and do not necessarily represent those of their affiliated organizations, or those of the publisher, the editors and the reviewers. Any product that may be evaluated in this article, or claim that may be made by its manufacturer, is not guaranteed or endorsed by the publisher.

## Footnotes

1
https://www.fil.ion.ucl.ac.uk/spm/

2
http://www.restfmri.net
